# Development and validation of a predictive model for estimating *EGFR* mutation probabilities in patients with non-squamous non-small cell lung cancer in New Zealand

**DOI:** 10.1186/s12885-020-07162-z

**Published:** 2020-07-14

**Authors:** Phyu Sin Aye, Sandar Tin Tin, Mark James McKeage, Prashannata Khwaounjoo, Alana Cavadino, J. Mark Elwood

**Affiliations:** 1 Epidemiology and Biostatistics, University of Auckland, B507, 22-30 Park Ave, Grafton, Auckland, 1072 New Zealand; 2 Pharmacology and Clinical Pharmacology, University of Auckland, Auckland, New Zealand; 3 Auckland Cancer Society Research Centre, University of Auckland, Auckland, New Zealand

**Keywords:** Non-small-cell lung carcinoma, Lung Cancer, Epidermal growth factor receptor, Mutation, Targeted therapy, Predictive models

## Abstract

**Background:**

Targeted treatment with Epidermal Growth Factor Receptor (EGFR) tyrosine kinase inhibitors (TKIs) is superior to systemic chemotherapy in non-small cell lung cancer (NSCLC) patients with *EGFR* gene mutations. Detection of *EGFR* mutations is a challenge in many patients due to the lack of suitable tumour specimens for molecular testing or for other reasons. *EGFR* mutations are more common in female, Asian and never smoking NSCLC patients.

**Methods:**

Patients were from a population-based retrospective cohort of 3556 patients diagnosed with non-squamous non-small cell lung cancer in northern New Zealand between 1 Feb 2010 and 31 July 2017. A total of 1694 patients were tested for *EGFR* mutations, of which information on 1665 patients was available for model development and validation. A multivariable logistic regression model was developed based on 1176 tested patients, and validated in 489 tested patients. Among 1862 patients not tested for *EGFR* mutations, 129 patients were treated with EGFR-TKIs. Their *EGFR* mutation probabilities were calculated using the model, and their duration of benefit and overall survival from the start of EGFR-TKI were compared among the three predicted probability groups: < 0.2, 0.2–0.6, and > 0.6.

**Results:**

The model has three predictors: sex, ethnicity and smoking status, and is presented as a nomogram to calculate *EGFR* mutation probabilities. The model performed well in the validation group (AUC = 0.75). The probability cut-point of 0.2 corresponds 68% sensitivity and 78% specificity. The model predictions were related to outcome in a group of TKI-treated patients with no biopsy testing available (*n* = 129); in subgroups with predicted probabilities of < 0.2, 0.2–0.6, and > 0.6, median overall survival times from starting EGFR-TKI were 4.0, 5.5 and 18.3 months (*p* = 0.02); and median times remaining on EGFR-TKI treatment were 2.0, 4.2, and 14.0 months, respectively (*p* < 0.001).

**Conclusion:**

Our model may assist clinical decision making for patients in whom tissue-based mutation testing is difficult or as a supplement to mutation testing.

## Background

Non-small cell lung cancer (NSCLC) comprises about 85% of all lung cancers. About 32.3% of NSCLC have mutation(s) of epidermal growth factor receptor (EGFR), ranging from 17.4% in Caucasian to 38.8% in Asian [1]. In addition to Asian ethnicity, *EGFR* mutations are well known for being more common among females and never smokers diagnosed with NSCLC [1, 2]. *EGFR* gene mutations associated with NSCLC occur in the tyrosine kinase domain (exons 18 to 21) and lead to constitutive activation of the EGFR tyrosine kinase [3]. Some constitutively activated mutant *EGFR* proteins are sensitive to EGFR tyrosine kinase inhibitor (TKI) drugs, such as those encoded by *EGFR* genes with exon 19 deletion mutations or exon 21 L858R point mutation, whereas others are not, such as those encoded by *EGFR* genes with exon 20 insertion mutations [3]. When first introduced into clinical use, EGFR-TKIs were approved for use for any patient with NSCLC without molecular selection [4]. Since then, several randomised trials have shown that NSCLC patients with activating *EGFR* gene mutations are responsive to EGFR tyrosine kinase inhibitors (EGFR-TKI) such as gefitinib and erlotinib [5–12]. A meta-analysis including seven trials showed that EGFR-TKIs resulted in prolonged PFS overall and in all subgroups compared to chemotherapy, with greater benefits in patients with exon 19 deletions, no smoking history and in female patients [13].

Testing for *EGFR* mutations has become a critical first step in personalised treatment of lung cancer. For several years now, clinical practice guidelines have recommended *EGFR* mutation testing for most patients with NSCLC, for individualising treatment and selecting patients for EGFR-TKI therapy [14–16]. These guidelines recommend against using demographic or clinicopathological factors for selecting patients for testing [14–16]. Not testing all eligible patients risks missing some patients with *EGFR* mutations, who will miss out on treatment with EGFR-TKIs and their well-known clinical benefits. Not testing also risks treating some patients without *EGFR* mutations with EGFR-TKIs, who have little or no chance of benefit. *EGFR* mutation testing methodologies have improved in recent times, for example, in their analytical sensitivity for detecting low levels of mutations in tissue specimens and body fluids, such as blood plasma and pleural effusions [17].

Despite clinical guidelines and improved methodologies for testing, the potential of personalised treatment of lung cancer for improving patient outcomes has not yet been fully realised in the setting of routine care. Testing rates remain low in many parts of the world, fuelled by sample limitations, funding constraints and selective testing referral practices. For example, our recent systematic review of studies from throughout the globe that had evaluated the utilisation of *EGFR* mutation testing in the setting of routine care, found that less than one third of a total of over 50,000 patients from 18 eligible studies were tested for *EGFR* mutations [18]. So, the implementation of *EGFR* mutation testing into routine clinical practice appears to have been less successful than might have been expected. Further effort will be required beyond aspirational guidelines and new testing methods to increase testing rates and appropriate use of EGFR-TKIs. To do so, estimation of pretest probability of *EGFR* mutations from universally available demographic factors has been suggested as a potential adjunct to mutation testing [19].

EGFR-TKIs became available in New Zealand from October 2010 [20]. *EGFR* gene mutation testing has been recommended in New Zealand for all NSCLC patients, except those with confidently diagnosed squamous cell carcinoma, since May 2013 [20]. Soon after testing had commenced in New Zealand, we began a population-based cohort study of non-squamous NSCLC patients presenting in northern New Zealand, which is on-going. Previously we reported on the uptake and impact of *EGFR* mutation testing in 1857 cohort patients diagnosed up until April 2014 [20]; *EGFR* mutation retesting of a subgroup of 532 cohort patients [21]; the impact of incomplete uptake of testing on estimates of mutation prevalence in 2701 cohort patients diagnosed up until December 2015 [22], and screening for ALK gene rearrangements in 3130 cohort patients diagnosed up until July 2016 [23]. In this large population-based study, in northern New Zealand, only 3.7% of non-squamous NSCLC patients were tested in 2010; this increased to 64.6% in 2014 and remained stable afterwards [20, 22]. These suboptimal testing rates were explained by selective referral practices and the lack of suitable tumour specimens being available for testing [20, 22]. *EGFR* mutation testing of plasma (liquid biopsy) offers one solution [24, 25] but it is prone to false negative test results, and it is expensive and not readily available in New Zealand. Thus, a good estimate of *EGFR* mutation probabilities would assist clinical decision making for treatment with EGFR-TKIs for patients with no test result available.

In a literature review up to Aug 2019, we identified nine *EGFR* mutation prediction models [26–34] that had been validated in an independent dataset. However, those studies were based on limited numbers of patients, confined to non-Asian patient populations, or included predictors that are routinely unavailable such as certain radiological features. The validity of these models in the New Zealand context is unknown, and may be more limited as New Zealand has diverse ethnic groups including Māori and Pacific people. Thus, we aimed to develop and validate a model based on the New Zealand patient data to estimate the probability of *EGFR* mutations in patients with non-squamous NSCLC. To do so, we further expanded our population-based retrospective cohort study to include a total of 3556 patients from northern New Zealand diagnosed with non-squamous NSCLC up until July 2017. Our analysis confirmed associations of *EGFR* mutations with gender, ethnicity and smoking status in a New Zealand context, and allowed us to develop and validate a statistical model for estimating the *EGFR* mutation probability, based on readily available demographic factors, in our local patient population.

## Methods

### Patient data

This population-based retrospective cohort study involved all patients who were diagnosed with non-squamous NSCLC and resident in northern New Zealand between 1 February 2010 and 31 July 2017. Patients were identified from the New Zealand Cancer Registry (NZCR), a well-established legally mandated population-based cancer registry that registers all primary cancers (excluding squamous and basal cell skin cancers) [35]. Following information was extracted: age, sex, ethnicity, District Health Board (DHB) region, date of diagnosis, morphology, site and disease extent. The data were linked to individual patient medical records (to obtain smoking data) and laboratory reports from TestSafe (to obtain *EGFR* mutation testing results). TestSafe is a clinical information sharing service, which compiles the laboratory and radiology reports from DHB facilities, community laboratories, and pharmacists [36]. *EGFR* mutations were tested by the Roche Cobas® real-time PCR that detects 41 variant sequences in the tyrosine kinase domain (exons 18–21) of the *EGFR* gene [37] or Agena MassARRAY OncoFOCUS™ [38] test that detects 128 *EGFR* gene mutations and 63 *KRAS*, *NRAS* and *BRAF* gene mutations, which we previously validated [21]. The positive *EGFR* mutation in this study refers to EGFR-TKI-sensitive mutations (i.e. exon 19 LREA deletion, L858R, G719X, S768I, L861Q, E709A and R776C) detected at diagnosis prior to EGFR-TKI therapy. Patients with *EGFR* mutations insensitive to gefitinib or erlotinib (exon 20 insertions, exon 20 T790M alone or those detected together with another sensitive mutation at diagnosis) were categorised as *EGFR* negative [39, 40].

### Data analysis

**Fig. 1:**
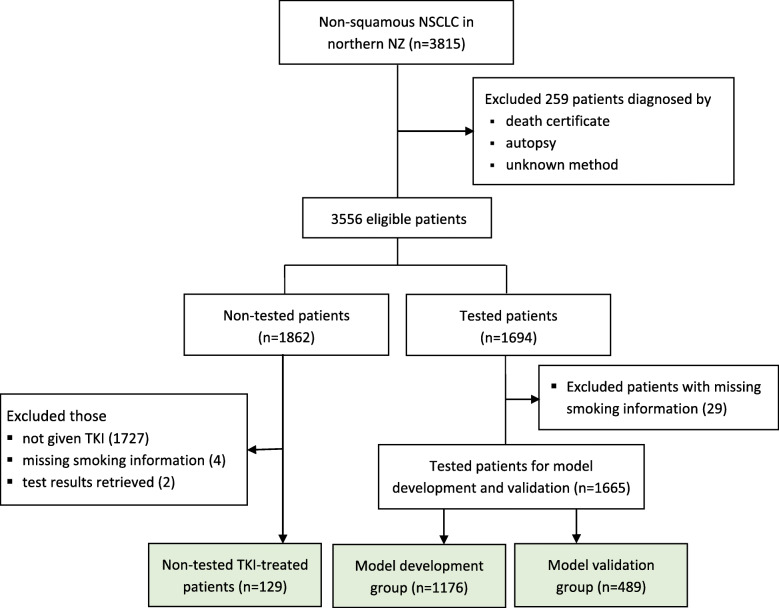
Flowchart showing the population-based retrospective cohort of patients diagnosed with non-squamous NSCLC in northern New Zealand between 1 January 2010 and 31 July 2017, and the groups of patients used in this study (coloured)

The data analysis was based on 1794 eligible (1665 tested, and 129 non-tested EGFR-TKI-treated) patients with complete data, derived from the total of 3815 patients (Fig. 1). The 1665 tested patients were divided into a development group (*n* = 1176), diagnosed from 1 Mar 2014 to 31 July 2017, which was used for model development and internal validation; and a validation group (*n* = 489), diagnosed from 1 Feb 2010 to 28 Feb 2014, which was used for external validation. A separate group of the 129 patients, who were not tested for the *EGFR* mutation but treated with EGFR-TKIs, was used to evaluate the model’s applicability. All analyses were performed using Stata v15. The model was then graphically illustrated in a nomogram by using the “regplot” command in R [41].

#### Model development

The model was developed in the development group of 1176 patients. First, single variable analyses were performed using age at diagnosis, sex, ethnicity, smoking status, disease extent and histology variables to identify the predictors of *EGFR* mutations. A *p*-value of < 0.05 was considered statistically significant. Then, a multivariable logistic regression analysis was used to estimate the probabilities of *EGFR* mutations. Age at diagnosis and extent of the disease were excluded from the model as they were statistically non-significant in multivariable regression. The histology variable, although significant, was omitted from the model since our patient sample included few patients with histological types other than adenocarcinoma; and the area under the curve (AUC) improved little by adding histology to the model. Thus, sex, ethnicity and smoking status were included in the final model. The resultant model was presented using a nomogram.

#### Model validation

The model was internally validated in the development group of 1176 patients and externally validated in the validation group of 489 patients [42], in terms of calibration and discrimination.

Calibration assesses the fit between predicted and observed mutation prevalence in groups of patients. To evaluate the model’s calibration, patients were divided into 5 groups created by the ranks of their predicted probabilities. Note that the numbers of observations in the groups were not equal as there were ties in predicted probabilities, that is, the same values were clustered into one group. Hosmer-Lemeshow’s goodness-of-fit tests were performed, and calibration was considered poor if the *p*-value was less than 0.05.

Discrimination assesses the model’s ability to distinguish between patients with a mutation and those without [42]. To evaluate the model’s discrimination, a Receiver Operating Characteristic (ROC) curve was plotted with the values of sensitivity (true positive rates) and 1-specifity (false positive rates) at consecutive cut points between 0 and 1 of the predicted probabilities. The area under the ROC curve (AUC) was used to determine the model’s performance in distinguishing between mutation-positive and -negative groups. An AUC of 1 represents perfect discrimination whereas 0.5 shows no discrimination beyond chance. The sensitivities and specificities were plotted against various predicted probability cut points, with the details reported for the cut points of 0.2 and 0.6.

#### Performance in untested patients

The applicability of the model was assessed in a group of 129 patients who were not tested for *EGFR* mutations, but were treated with EGFR-TKIs. The validity of the model is shown by differences in treatment outcomes in terms of predicted mutation status, in the absence of tissue testing. Patients were categorised into three mutation probability groups using the cut points of 0.2 and 0.6. Overall survival and proportions remaining on EGFR-TKI over time up to 3 years were then compared using Kaplan-Meier estimates and log-rank tests. Overall survival was measured from the start of EGFR-TKI to the date of death, and surviving patients were censored on 31 May 2018. Time on EGFR-TKI treatment was measured from the start date to the stop date of the treatment or date of death.

## Results

### Patient characteristics

A total of 3815 potentially eligible patients from northern New Zealand were identified who had been diagnosed with non-squamous NSCLC between 1 January 2010 and 31 July 2017 (Fig. 1). Patients whose diagnoses were made by death certificate, autopsy or an unknown basis were excluded (*n* = 259). Of 3556 eligible patients, 1862 patients were not tested for *EGFR* mutations including 129 patients who were treated with EGFR-TKIs. Of the 1694 patients who were tested for *EGFR* mutation(s), 29 were excluded due to missing smoking information. Of the remaining 1665 tested patients, 342 (20.5%) were mutation-positive (21% in the development group and 18% in the validation group) (Table 1). Of 339 *EGFR* mutation-positive patients, 164 (48.4%) had exon 19 deletions, 137 (40.4%) had L858R point mutations and 38 (11.3%) had other mutations (Table 1). Thirty-seven patients (exon 20 insertions, *n* = 33; exon 20 T790M alone, with exon 21 L858R or exon 19 deletion, *n* = 4) were categorised as *EGFR* mutation-negative. The distribution of demographic, clinical and pathological factors was similar between the development, validation and non-tested EGFR-TKI-treated groups. A majority of patients were between 50 and 79 years old, predominantly female, NZ European, ex-smokers, and had distant spread of the disease at diagnosis. Most tumours were adenocarcinoma (Table 1).

**Table 1:** Patient characteristics of the development, validation and non-tested EGFR-TKI-treated groups

	Development group		Validation group		Total		Non-tested EGFR-TKI treated group	
	N	%	N	%	N	%	N	%
Total	1176	100	489	100	1665	100	129	100
Mutation status								
No	927	78.8	399	81.6	1326	79.6	–	–
Yes	249	21.2	90	18.4	339	20.4		
Mutation types								
Exon 19 deletion Exon 21 L858R Exon 18 G719X Exon 18 G719X + Exon 20 S768I Exon 20 S768I Exon 20 S768I + Exon 21 L858R Exon 18 G719X + Exon 18 E709A Exon 21 L861Q Exon 20 R776C + Exon 21 L858R Exon 18 G719X + Exon 21 L861Q Exon 19 deletion + Exon 20 S768I	117 102 12 7 2 3 2 1 1 1 1	47.0 41.0 4.8 2.8 0.8 1.2 0.8 0.4 0.4 0.4 0.4	47 35 3 3 1 0 0 1 0 0 0	52.2 38.9 3.3 3.3 1.1 0 0 1.1 0 0 0	164 137 15 10 3 3 2 2 1 1 1	48.4 40.4 4.4 3.0 0.9 0.9 0.6 0.6 0.3 0.3 0.3	–	–
Age at diagnosis								
< 50 yr	74	6.3	40	8.2	114	6.9	15	11.6
50–59 yr	186	15.8	92	18.8	278	16.7	36	27.9
60–69 yr	361	30.7	167	34.2	528	31.7	46	35.7
70–79 yr	412	35.0	144	29.5	556	33.4	29	22.5
> =80 yr	143	12.2	46	9.4	189	11.4	3	2.3
Sex								
Male	513	43.6	218	44.6	731	43.9	54	41.9
Female	663	56.4	271	55.4	934	56.1	75	58.1
Ethnicity								
NZ European	682	58.0	293	59.9	975	58.6	75	58.1
NZ Maori	175	14.9	68	13.9	243	14.6	20	15.5
Pacific	127	10.8	53	10.8	180	10.8	13	10.1
Asian	177	15.1	68	13.9	245	14.7	20	15.5
Other & Unknown	15	1.3	7	1.4	22	1.3	1	0.8
Smoking								
Current smoker	264	22.5	112	22.9	376	22.6	29	22.5
Non-smoker	308	26.2	116	23.7	424	25.5	41	31.8
Ex-smoker	604	51.4	261	53.4	865	52.0	59	45.7
Extent								
Localised	130	11.1	37	7.6	167	10.0	2	1.6
Adjacent or regional	266	22.6	120	24.5	386	23.2	22	17.1
Distant	561	47.7	232	47.4	793	47.6	80	62.0
Unknown	219	18.6	100	20.5	319	19.2	25	19.4
Histology								
Adenocarinoma	1024	87.1	433	88.6	1457	87.5	105	81.4
Other	152	12.9	56	11.5	208	12.5	24	18.6

### The predictive model for estimating the probability of EGFR mutation

**Fig. 2:**
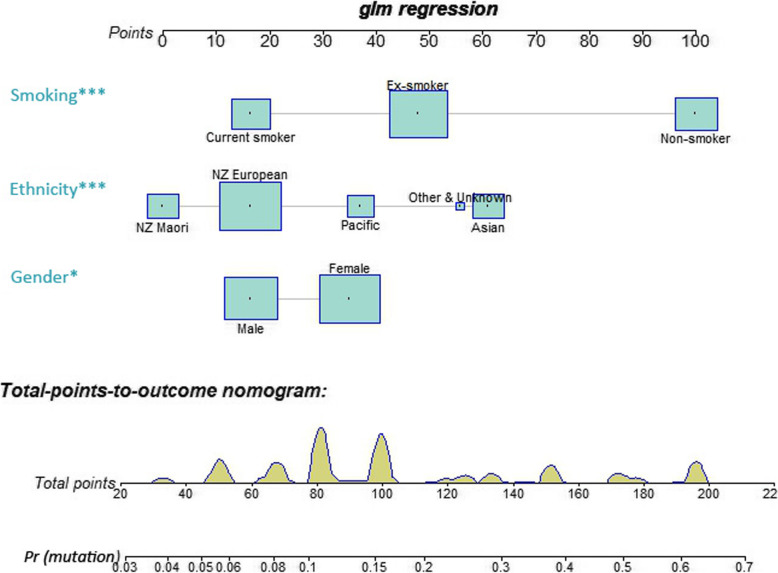
Nomogram of the EGFR mutation predictive model. The predictors are arranged based on their effect size. Asterisks refer to the levels of statistical significance: *p < 0.05, ***p < 0.001. The square boxes show the distribution of the data. The points for each predictor are observed by drawing a perpendicular line towards the points bar at the top of the nomogram, and are summed to obtain a total score. The estimated probability of mutation positivity is provided in correspondence to the total score

In single factor analyses, sex, ethnicity, smoking status, disease extent and histology were significantly associated with the *EGFR* gene mutation status (Table 2). In the final multivariable model including sex, ethnicity and smoking status, females (compared to males; OR = 1.5, 95% CI 1.1–2.1), Asian and Pacific patients (compared to European patients; OR = 2.8 and 1.6, respectively) and non-smokers and ex-smokers (compared to current smokers; OR = 6.7 and 2, respectively) were more likely to harbour *EGFR* mutation(s) (Table 2). The nomogram illustrates the predictive model with the estimated *EGFR* mutation probabilities (Fig. 2).

**Table 2:** Single and multi-variable analysis

	Single factor analysis			Multivariable analysis		
	Mutation positive		*p*-value	OR	(95% CI)	*p*-value
	N	%				
Total	249	21.17				
Age at diagnosis			0.063			
< 50 year	21	28.38				
50–59 year	37	19.89				
60–69 year	60	16.62				
70–79 year	97	23.54				
> =80 year	34	23.78				
Sex			< 0.001			
Male	77	15.01		1		
Female	172	25.94		1.5	(1.1–2.1)	0.014
Ethnicity			< 0.001			
NZ European	105	15.4		1		
NZ Maori	16	9.14		0.7	(0.4–1.2)	0.201
Pacific	35	27.56		1.6	(1.0–2.6)	0.052
Asian	87	49.15		2.8	(1.8–4.2)	< 0.001
Other & Unknown	6	40		2.5	(0.8–7.5)	0.118
Smoking status			< 0.001			
Current smoker	19	7.2		1		
Ex-smoker	85	14.07		2	(1.2–3.5)	0.008
Non-smoker	145	47.08		6.7	(3.9–11.7)	< 0.001
Extent			0.005	–		
Localised	42	32.31				
Adjacent or regional	50	18.8				
Distant spread	106	18.89				
Unknown	51	23.29				
Histology			< 0.001	–		
Adenocarcinoma	238	23.24				
Other	11	7.24

### Calibration of observed and predicted probabilities

**Fig. 3:**
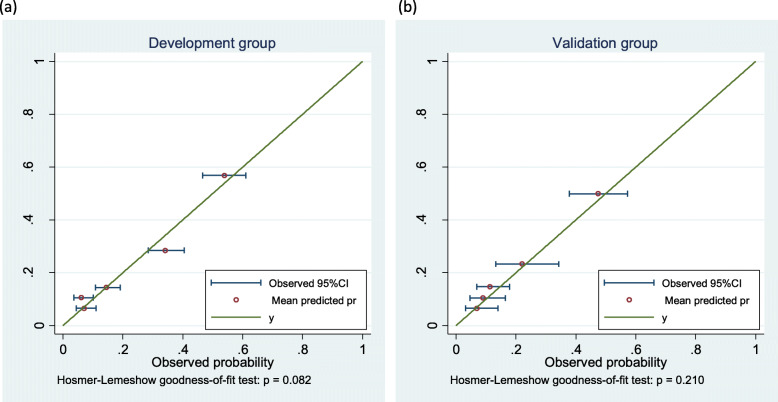
Calibration plots. Assessment of the model’s internal validity using the development group (**a**), and external validity using the validation group (**b**): the mean predicted *EGFR* mutation probabilities plotted against the observed mutation probabilities with their 95% CI, shown in five groups created by the ranks of the predicted probabilities. Hosmer-Lemeshow test compares the observed and predicted probabilities: a *p*-value of > 0.05 indicates good calibration

In both development and validation groups, the predicted probabilities ranged from 4 to 62% (Table 3, Fig. 3). The mean predicted probabilities fell within the 95% confidence intervals of observed probabilities for all groups. The Hosmer-Lemeshow test showed adequate goodness-of-fit of the model both in the development group (*p* = 0.08), and in the validation group (*p* = 0.21).

**Table 3:** Calibration assessment of the *EGFR* mutation predictive model

Group ^a^	N	Predicted *EGFR* mutation			Observed *EGFR* mutation		
		Number	Mean	(min-max)	Number	Proportion	(95% CI)
Development group							
1	254	17	0.07	(0.04–0.08)	18	0.07	(0.04–0.11)
2	228	24	0.11	(0.09–0.11)	14	0.06	(0.04–0.10)
3	282	41	0.14	(0.11–0.15)	41	0.15	(0.11–0.19)
4	234	67	0.28	(0.16–0.38)	80	0.34	(0.28–0.40)
5	178	101	0.57	(0.39–0.62)	96	0.54	(0.47–0.61)
Validation group							
1	100	7	0.07	(0.04–0.08)	7	0.07	(0.03–0.14)
2	100	10	0.10	(0.09–0.11)	9	0.09	(0.05–0.16)
3	133	20	0.15	(0.11–0.15)	15	0.11	(0.07–0.18)
4	59	14	0.23	(0.16–0.29)	13	0.22	(0.13–0.34)
5	97	48	0.50	(0.31–0.62)	46	0.47	(0.38–0.57)

^a^ The five groups were created by the ranks of the predicted probabilities

### Discrimination between mutation positive and negative patients

**Fig. 4:**
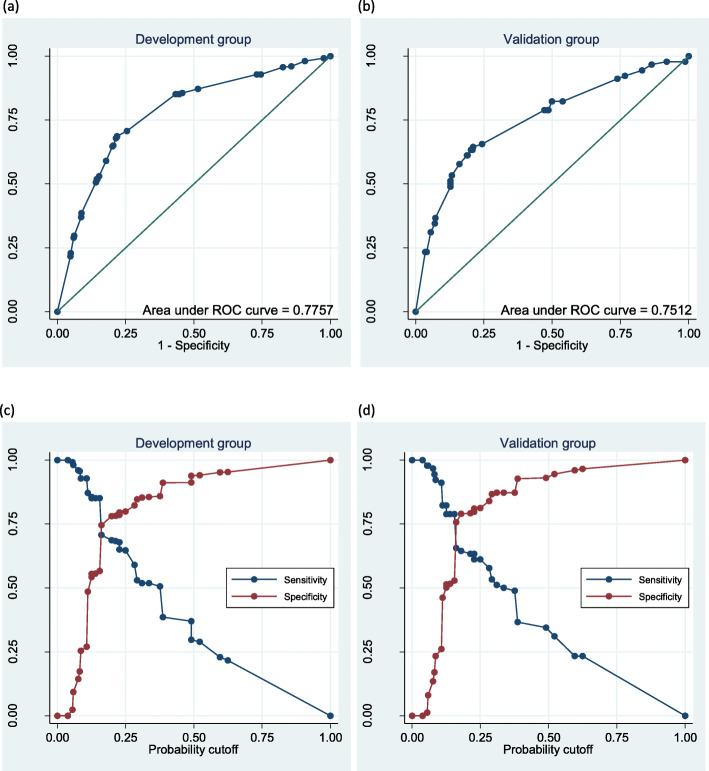
Sensitivity and specificity reports. ROC curves using the development group (**a**), and the validation group (**b**); Detailed sensitivity & specificity report for individual cut-points using the development group (**c**), and the validation group (**d**)

The Receiver Operating Characteristic (ROC) curves show the probability curves with corresponding true positive rates and false positive rates (Fig. 4). The model’s AUC was similar in the development group (0.78) and the validation group (0.75). The maximum separation was at probability cut point of 0.2, achieving a negative predictive value (NPV) of 90% for the development group and 91% for the validation group; a positive predicted value (PPV) of 46 and 41%; and an Informedness index of 0.46 and 0.43, respectively (Table 4). An NPV of 90% means that 90% of patients classified by the model as not having *EGFR* mutations at this cut point, in actuality did not have an *EGFR* mutation. A PPV of 46% means that 46% of patients classified by the model as having *EGFR* mutation, in actuality had an *EGFR* mutation. An Informedness index of 0.46 means an appropriate use of information [43].

**Table 4:** Detailed sensitivity and specificity report for *EGFR* mutation predicted probability cut-points of 0.2 and 0.6

	Development group		Validation group	
	0.2	0.6	0.2	0.6
Sensitivity	68.27%	21.69%	63.33%	23.33%
Specificity	78.21%	95.25%	79.20%	96.49%
Positive predictive value	45.70%	55.10%	40.71%	60.00%
Negative predictive value	90.17%	81.91%	90.54%	84.80%
Informedness index ^**a**^	0.46	0.17	0.43	0.2

^a^ Informedness index is calculated as sensitivity+specificity-1. Interpretation: 0 means the test is useless, 1 means the test is perfect, and a value of > 0 means an appropriate use of information [*Reference*: Youden WJ. Index for rating diagnostic tests. Cancer. 1950;3(1):32–5]

### Treatment outcomes by predicted mutation probability in a non-tested EGFR-TKI-treated group

**Fig. 5:**
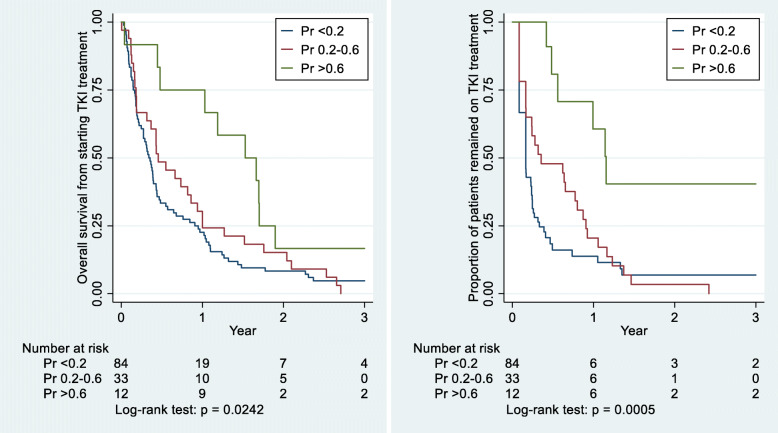
Survival outcomes from EGFR-TKI treatment in a group of untested NSCLC patients (*n* = 129) by estimated *EGFR* mutation probability (pr < 0.2, 0.2–0.6, and > 0.6)

This group involves 129 patients treated with EGFR-TKIs, who were not tested for *EGFR* mutations. Figure 5 shows that outcomes are related to the estimated probability of a mutation as given by the model. Using the 0.2 and 0.6 cut points, the median overall survival times from starting EGFR-TKI treatment were 4 months in < 0.2 group, 5.5 months in 0.2–0.6 group, and 18.3 months in > 0.6 group (*p* = 0.024). The median times on EGFR-TKI treatment from the start date were 2 months, 4.2 months, and 14 months, respectively (*p* < 0.001).

## Discussion

We developed a model to estimate the probability of *EGFR* mutation based on a population-based series of 1176 non-squamous NSCLC patients in northern New Zealand. Our model included three predictors that were significantly associated with the *EGFR* mutation status in the multivariable analysis: sex, ethnicity and smoking status. The female sex, Asian ethnicity and being a non-smoker were highly associated with higher prevalence of *EGFR* mutation, as observed in previous studies [1, 2].

We presented the fitted model using a nomogram, which is an increasingly used format for clinical prediction models for its ability to provide exact predictions [44]. We validated the model using established performance measures [44]. The model showed good calibration with the mean predicted probabilities being within the 95% limits of the observed values in all the groups for both development and validation. The goodness-of-fit was slightly better in the validation group than the development group. The AUCs of 0.78 in the development group and 0.75 in the validation group inferred that our model performed reasonably well. Further, in a retrospective group of NSCLC patients treated with EGFR-TKIs without *EGFR* mutation testing, patients with higher *EGFR* mutation probabilities estimated from the model had significantly longer overall survival and longer duration of EGFR-TKI treatment than those with lower *EGFR* mutation probabilities.

We considered possible limitations of our model. The patients included in our model were of necessity those who had been tested for the *EGFR* gene mutation. Our earlier work showed that *EGFR* mutation testing increased from 3.7% of all patients in 2010 to 64.6% in 2014 in this population-based retrospective cohort [20]. In parallel, recorded *EGFR* mutation rates decreased from 43.8% in 2010 to 16.8% in 2014, reflecting decreases in selective testing [22]. Taking into account this variation, we assessed the external validity of the model in the independent earlier period dataset, and the results were similar to those in the development group. The *EGFR* mutation prevalence in this study is within the range of the largest systematic review, being 47% in Asia-Pacific region and 12% in Australia [2]. The predictive model does not provide information about what particular *EGFR* mutation may be present, which could be important for clinical decision-making.

Models with combined clinical factors and imaging features may improve performance in predicting *EGFR* mutation status [26, 28, 33, 45–48]. However, extracting radiological features from clinical or radiological reports is complex unless a particular recording system is added to routine records for this purpose. For instance, in Zhang et al. [28] study, as many as 485 CT features were used for their Rad_signature scoring system, which is unlikely to be feasible in our setting. Thus, we developed the current model with the important available clinical factors only.

Our model includes New Zealand specific ethnicities including Māori and Pacific people. Māori and Pacific people have a higher incidence of lung cancer and poorer survival, compared to the New Zealand European population [49]. But, the testing rate was particularly low in Māori patients compared to other ethnic groups [22]. Our model may be helpful in addressing ethnic disparity in lung cancer patients in New Zealand. Moreover, a combined nomogram for both Asian and non-Asian populations showed unsatisfactory accuracy in the study of Gevaert et al. [26]. It claimed that Asian patients had substantially different distributions of the predictors. Thus, developing ethnic specific models may be relevant in future research.

We categorised the patients into three groups based on the probability of *EGFR* mutation positivity: low (< 0.2), medium (0.2–0.6) and high (> 0.6) probability groups. We then compared the duration of benefit and the overall survival from the start of EGFR-TKI treatment between the three probability subgroups in a group who had been treated with EGFR-TKIs second-line, without a tissue test result for mutations. The outcomes were significantly more favourable in the higher probability group than the lower probability group with outcomes of the medium probability group being intermediate of the other two. These findings demonstrate that our model has the potential to predict mutation status and can differentiate between untested patients who have good outcomes from EGFR-TKI treatment and those who will have poor treatment outcomes. Thus, when testing is not possible, those in the high probability group could be considered for EGFR-TKI treatment. Conversely, those in the low probability group should not receive an EGFR-TKI. These findings are consistent with published randomised controlled clinical trials showing the relative benefits of EGFR-TKIs versus chemotherapy for untested NSCLC patients to critically depend upon the proportion of patients demonstrated to have *EGFR* mutations by post hoc mutation testing [6, 7, 50–52].

*EGFR* mutation status can also be estimated by liquid biopsy to detect circulating DNA in plasma. The sensitivity of this, compared with tissue biopsy, varies considerably in different series and with the methods used, but may be about 85% in advanced disease, but lower in less advanced cases [24, 25]. However, these methods are expensive and not readily available in New Zealand. False negative results are of concern. While our *EGFR* mutation predictive model cannot replace molecular testing, in patients for whom tissue biopsy is difficult, it could be used in conjunction with liquid biopsy, giving further attention to patients with a high estimated probability, but a negative liquid biopsy result, suggesting a false negative.

Our study is moderate in size, and applies to a multi-ethnic population in New Zealand, so application to other populations requires further studies. Our model used only three factors, and other factors such as radiological appearances, blood markers such as CEA [53], or more precise classification of smoking history, may yield improved models.

## Conclusion

We have developed and validated a model for estimating the probability of *EGFR* mutations in non-squamous NSCLC patients based on routinely collected factors. This model may be useful for supporting clinical decisions for patients in whom mutation testing is difficult or for use alongside mutation testing.

## Data Availability

The data that support the findings of this study are available from New Zealand Cancer Registry and TestSafe information sharing database but restrictions apply to the availability of these data, and so are not publicly available. Data are however available from the authors upon reasonable request and with permission of corresponding data owners.
